# Digitalis in Cardiac Failure

**Published:** 1906-02-17

**Authors:** 


### DIGITALIS IN CARDIAC FAILURE.

Taking as a text a patient showing the familiar evidences of failing compensation in cardiac valvular disease, Dr. J. Mitchell Bruce [1] offers a number of practical suggestions on the use of digitalis. First he emphasises the fact that the diuresis produced by digitalis in ordinary doses of the pharmacopœial preparations does not make its appearance for several days. Hence follow two important conclusions. First, the drug must not be hastily abandoned because there is no manifest diuretic influence in the course of a day or two; and secondly, while waiting for the slower action of digitalis such remedies as caffeine, ether, ammonia, and strychnine must be employed in order to carry the patient over the preliminary "period of suspense." A second lesson in the use of digitalis in cardiac failure is that the drug must be given freely. In the case under discussion 10 minims of the tincture every four hours produced no appreciable effect even at the end of seven days; but when the dose was increased to 15 minims free diuresis promptly followed. A parallel statement may be made in regard to the dosage of digitalis in dealing with cardiac irregularity. It not infrequently happens that when a full dose of the tincture, say 10 minims, has been given at intervals of four hours or so for several days, and the pulse continues small, irregular and frequent, the suggestion arises that the conditions may be due to a toxic action of the remedy. But the acceleration and irregularity of the heart, which are described in the text books as expressions of the toxic action of digitalis, do not occur clinically. Hence so far from being deterred from persistence in the use of digitalis by a small and irregular pulse, the rule in such circumstances is, not to stop the remedy, but to increase the dose. This policy in the case under discussion was followed by a reduction in the pulse rate to 50 when, more especially in the presence of aortic regurgitation, it was decided to reduce the tincture from 15 to 5 minims. Even this seemed to be sufficient, when the action had been once established, to keep the rate of the heart under 60 per minute, and thus on the twenty-second day of treatment the digitalis was stopped, and reliance was placed on nux vomica alone. This was shortly followed by a return of the former symptoms, including small, rapid, and irregular pulse, dropsy, and scanty urine. Upon this phase of the record it was pointed out that though the action of digitalis on the heart persists for days after its cessation, the drug must be continued for a certain length of time if the benefits it has effected are to be maintained. The rule should be to reduce slowly the dose of the drug after relieving the urgent manifestations of cardiac failure. In this particular instance the rule was departed from because the pulse had fallen to a very low rate, and the long diastolic pause and aortic reflux caused some degree of alarm. But the event proved that the sudden reduction of the dose and its early suspension were errors, as shown by return of the symptoms. On further prescription of the remedy its remedial value was again demonstrated, and once again the full effect was only manifested after the lapse of several days and under the influence of full doses of the tincture. Another lesson enforced by this record is the unwisdom of the statement that digitalis should not be given in aortic regurgitation. If there is cardiac failure the case is one for digitalis, and the progress of the case or the influence of the remedy find their most unequivocal expression in the quantity of the urine passed.

[1] Brit. Med. Jour., Jan. 6, 1906.

